# Evolution of the Clinical, Dermoscopic and Pathologic Diagnosis of Melanoma

**DOI:** 10.5826/dpc.11S1a163S

**Published:** 2021-07-01

**Authors:** Harald Kittler

**Affiliations:** Department of Dermatology, Medical University of Vienna, Vienna, Austria

**Keywords:** Melanoma, dermoscopy, dermatoscopy, pathology

## Abstract

The conventional narrative states that the steadily rising incidence of melanoma among fair-skinned Caucasian populations during the last decades is caused by excessive UV-exposure. There is, however, no doubt that other factors had a significant impact on the rising incidence of melanoma. Pre-1980s the clinical diagnosis of melanoma was based on gross criteria such as ulceration or bleeding. Melanomas were often diagnosed in advanced stages when the prognosis was grim. In the mid-1980s education campaigns such as the propagation of the ABCD criteria, which addressed health care professionals and the public alike, shifted the focus towards early recognition. Dermatoscopy, which became increasingly popular in the mid-1990s, improved the accuracy for the diagnosis of melanoma in comparison to inspection with the unaided eye, especially for flat and small lesions lacking ABCD criteria. At the same time, pathologists began to lower their thresholds, particularly for the diagnosis of melanoma in situ. The melanoma epidemic that followed was mainly driven by an increase in the number of in situ or microinvasive melanomas. In a few decades, the landscape shifted from an undercalling to an overcalling of melanomas, a development that is now met with increased criticism. The gold standard of melanoma diagnosis is still conventional pathology, which is faced with low to moderate interobserver agreement. New insights in the molecular landscape of melanoma did not translate into techniques for the reliable diagnosis of gray zone lesions including small lesions. The aim of this review is to put our current view of melanoma diagnosis in historical context and to provide a narrative synthesis of its evolution. Based on this narrative I will provide suggestions on how to rebuild the trust in melanoma diagnosis accuracy and in the benefit of early recognition.

## Introduction

A conventional introduction of an article on melanoma diagnosis usually includes statements on the rising incidence and mortality of melanoma in general, and that melanoma is the most lethal type of skin cancer. While the latter is not true (the lethality of Merkel cell carcinoma is higher), the incidence and mortality rates of melanoma seem to have peaked recently[1]. Furthermore, a conventional introduction will also include a statement that the steadily rising incidence of melanoma among fair-skinned Caucasian populations is caused by excessive intermitted UV-exposure[2–4], but will not mention that increased public awareness, early recognition campaigns, technical innovations, and lower thresholds of pathologists had a significant impact on the rising incidence of melanoma.

A Pubmed literature search using “melanoma” and “diagnosis” keywords yields 68102 articles if unrestricted and 9621 articles if the search is limited to the past 3 years. It is obvious that even the most ambitious review cannot cover all aspects of melanoma diagnosis. Like any type of scientific research, a review should not only collect data but also create a narrative with explanatory power. The aim of this review is not to be exhaustive but to focus on the evolution of the criteria and concepts for melanoma diagnosis. While melanoma was exceedingly rare pre-1980, we observed a dramatic increase in the incidence in some parts of the world [5,6]. This statement deserves an explanation. It is the major underlying hypothesis of this review that this epidemic can in most parts be explained by changing diagnostic concepts and progress in the field of in-vivo examination techniques. This interpretation is increasingly shared by others, although with different conclusions [7]. Advocates of early recognition, dermatologists and general practitioners alike, are faced with increasing criticism [8,9].

According to the opinions of critics, the increased incidence of melanoma is due to overdiagnosis, that goes hand in hand with an increase in the number of unnecessary biopsies and excisions driven by in vivo examination techniques such as dermatoscopy. In this scenario overdiagnosis and unnecessary surgery lead, according to critics, to increased morbidity and anxiety, while at the same time there is no evidence supporting improved survival following early recognition.

As a solution, Welch et al recently suggested not to biopsy pigmented lesions with a diameter smaller than 6 mm [7]. While Welch et al rightly addressed many problematic issues in the field of melanoma diagnosis, this suggestion indicates a lack of knowledge of and a lack of confidence in current diagnostic techniques.

### Clinical Diagnosis

By clinical diagnosis we refer to the diagnosis of melanoma with the unaided eye, which was state-of-the art before the introduction of the dermatoscope. The natural starting point for a review on the clinical diagnosis of melanoma are the ABCD criteria. These were popularized in the mid-1980s, mainly in the US [10]. Before the 1980s the diagnosis of melanoma was based on gross features such as ulceration or bleeding. The ABCD criteria mark the first attempt to summarize melanoma criteria in a simple mnemonic that is easy to remember. It includes asymmetry (A), border irregularity (B), color variegation (C), and diameter larger than 6mm (D) criteria. The ABCD criteria were developed following the increasing need to educate physicians and the public to recognize melanoma at earlier stages. In the words of Darrel Rigel, who was part of the team that popularized the ABCD criteria in the 1980s, it was *“intended to be a simple tool that could be implemented in daily life, a mnemonic as easy as ABC to alert both laypersons and healthcare professionals to the clinical features of early melanoma”* [11]*.*

Along the same line, Rona McKie propagated a 7-point checklist to support non-dermatologists in recognizing possible melanomas[12,13]. The checklist was known as the Glasgow 7-point checklist and was quite popular in the UK. The clinical ABCD criteria and the Glasgow 7-point checklist became blueprints for other simple mnemonics, such as the ABCD rule [14] or the 7-point checklist for dermatoscopy [15], which even in terms of their naming, directly refer to their historic models. Interestingly, neither the ABCD criteria nor the Glasgow 7-point checklist were derived from statistical evidence but rather from the best judgements of expert clinicians. At the same time in 1985, A Bernard Ackerman, who was an influential figure in the field of dermatology and dermatopathology, wrote a lively plea for early recognition of melanoma entitled “No one should die of malignant melanoma” [16]. In a series of articles and book chapters, Ackerman and his coworkers set forth and refined criteria for the clinical and histopathologic diagnosis of melanoma in situ. Pre 1980s, the recognition of melanoma in situ was not widely accepted and it was rather viewed as a precursor but not as authentic melanoma. The combined effect of increased public awareness and education of healthcare professionals to recognize the early stages of melanoma had a major impact on the diagnosis of melanoma. The incidence of melanoma increased, and the epidemic of melanoma started.

From an early recognition point of view, the most critical parameter in the ABCD criteria was the diameter. A size threshold puts a limit to how early melanomas can be diagnosed. The ABCD rule gives credit to the fact that small melanomas are difficult to diagnose because melanomas smaller than 6 mm are usually not asymmetric and multicolored, at least when viewed with the unaided eye. Size limits were also part of other algorithms. The Glasgow 7-point checklist established a size limit of 7 mm. A popular algorithm for the diagnosis of acral melanoma developed by Saida et al determined a size limit of 7 mm for the diagnosis of acral melanoma [17]. The diagnosis of melanoma of the nail matrix is discouraged if the pigmentation covers less than 1/3 of the nail plate [18]. Size limits have the problem that, at least in theory, all melanomas start smaller than 6 mm. A reevaluation of the ABCD criteria in 2004, however, concluded that the size limit of 6 mm should not be lowered[19]. In light of the fact that melanomas smaller than 6 mm were increasingly recognized, the authors suggested that *“the ABCD should be expanded to ABCDE (E standing for enlargement or evolution) to emphasize the significance of evolving pigmented lesions for the diagnosis of melanoma”.*

The disadvantage of the newly added E criterion relies on the fact that it depends on information collected over time. Although the self-reported history of patients or information provided by a spouse or partner can at times be a valuable source for this type of information, it is not perfectly reliable [20, 21]. Total body photography (TBP) on the other hand, helps to detect new and changing lesions independent from the attention of the patient and thereby facilitates the detection of small and inconspicuous melanomas [22–24]. It also reduces the number of unnecessary excisions of benign lesions [25]. In a recent meta-analysis, Ji-Xu calculated that total body photography of high-risk individuals significantly reduced the number of biopsies needed to detect one melanoma from 14.8 to 8.6 [26]. Most melanomas detected by TBP were in situ, highlighting the impact of TBP for early recognition. TBP is especially useful for individuals with multiple nevi, in whom melanomas are more difficult to detect because of the abundance of nevi [27].

The “ugly duckling” approach addresses this difficulty in attempting to find the one outlier among multiple similar looking lesions. The first attempts to popularize the “ugly duckling” approach can be attributed to the French dermatologist JJ Grob, who co-authored an article on this topic in 1998 [28]. Unlike the ABCD criteria, the “ugly duckling” method is a comparative approach that takes into account the landscape of nevi in a particular patient. It tacitly assumes that individuals have a nevus archetype and that deviations from this archetype may indicate malignancy. It is an informal method as there is no rigorous definition regarding the kind of deviation that is significant. All kinds of deviation have been used to identify the outlier lesion, such as a pink lesion among pigmented lesions, a large lesion among small lesions, and a chaotic lesion among symmetric lesions. This approach can also be used to increase specificity in patients with multiple “atypical” nevi. If all nevi look atypical and none is standing out the significance of “atypia” decreases. This inverse interpretation of the “ugly duckling” approach has been more formally investigated in the field of dermatoscopy by Argenziano and coworkers [29]. In 2021, Soenksen et al. successfully used the “ugly duckling” approach to automatically detect outlier lesions from photographic overviews with artificial intelligence (AI) [30].

### Dermatoscopy

**Figure 1:**
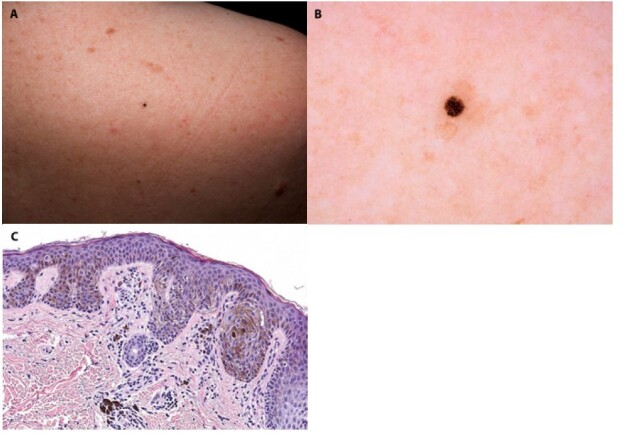
(A) Clinical, (B) dermatoscopic, (C) histopathologic view of a tiny melanoma (<3 mm).

The seminal paper of Pehamberger on pigmented skin lesions pattern analysis, published in 1987 [31] paved the way for future developments of the dermatoscopic diagnosis of melanoma. It described patterns of benign and malignant pigmented skin lesions and introduced and defined dermoscopic criteria that are still used today. A closer look at this classical article, however, reveals that the melanomas shown in the figures are large and could have been diagnosed without dermatoscopy. The method of dermatoscopy was still evolving and the world of dermatology was not ready to accept that melanomas can be small (smaller than 6 mm) and inconspicuous (Figure 1). Soon thereafter other groups followed and presented their own interpretation of pattern analysis. Old concepts such as the ABCD criteria and the Glasgow 7-point checklist were reused for dermoscopy. Stolz et al invented the ABCD rule of dermoscopy and Argenziano et al the 7-point checklist[14,15]. Both methods aimed to differentiate melanomas from nevi. Other noteworthy algorithmic approaches include Menzies rule [32], the CASH algorithm [33], and the chaos and clues method, which appeared later [34]. Over the years 3 meta-analysis showed how dermatoscopy improved diagnostic accuracy for melanoma, compared to an unaided eye inspection [35–37]. Another milestone was the Second Consensus Conference of Dermoscopy, which was virtually held [38]. This was a turning point for the evolution of dermatoscopy because it marks the beginning of a fruitful international collaboration among different groups that tried to establish a consensus for criteria and terminology. Prior to this milestone event, the study of dermatoscopy was fragmented into different small research groups that often antagonized each other.

In the following years dermatoscopy differentiated into a complex science and criteria for melanoma were refined. Special criteria were described for acral melanoma [39–41], facial melanoma [42–44], amelanotic and hypomelanotic melanomas [45,46], nodular melanomas [47,48], mucosal melanoma[49], nail matrix melanoma [50–53], and melanomas on chronic sun damaged skin [54]. Smaller and smaller lesions were identified as melanomas thanks to dermatoscopy pushing the diagnostic boundaries, also in dermatopathology [54–58]. Furthermore, Argenziano et al demonstrated that when applied by experienced users, dermatoscopy reduces the number of biopsies or excisions needed to detect 1 melanoma [59]. Despite these advancements, it became clear that dermatoscopy had its limitations [59–63]. It was reported that some small and flat melanomas lack melanoma clues at the beginning and can only be diagnosed by observing changes over time with sequential digital dermatoscopy [64–68]. The finding was immediately criticized as just another way to inflate the melanoma epidemic [69]. The introduction of sequential dermatoscopy to recognize changes over time, mirrors the letter E (for evolving) addition to the ABCD criteria.

Finally, dermatoscopic images are increasingly used for training of machine learning algorithms [70–75]. Computer algorithms based on deep learning outperformed dermatologists in some studies and increased the expectations that AI will replace human expertise, at least for some applications such as teledermatoscopy. The expectations are likely exaggerated because AI-based algorithms still lack the kind of adaptive general knowledge that is necessary to act independently from humans. It is likely, though, that AI will transform images-based diagnostic medicine in many ways. As recently demonstrated by Tschandl et al, collaboration between humans and computers is more promising than competition [72].

### Histopathologic Diagnosis

While it is easy to pin down the beginning of the clinical and dermatoscopic diagnosis of melanoma evolution, the same does not apply to histopathology. A possible choice is the work of LV Ackerman in the late 1940s. It was one of the first to systematically describe the pathology of “melanocarcinoma”, as defined then [76]. All clinical photos and micrographs in his original publication of a series of 75 cases show advanced cases of melanoma. Of 40 patients who underwent dissection of the local lymph nodes, 37 already had lymph node metastasis at the time of diagnosis. The article is mostly interesting for its summary of beliefs about melanoma prevalent in those days. According to LV Ackerman melanoma usually starts in a mole and “*it is most unusual to find the changes of malignant melanoma entirely within the epidermis with no change in the dermis.”* Interestingly, among the suggested treatments mentioned in this article there was also castration, because it was believed that hormones have an impact on the course of the disease. In 1953, A Allen and S Spitz co-authored an article, in which they set forth their belief that all melanomas start in a preexisting mole, especially in a so called “*active junctional nevus*” [77]. The micrograph of the *“activated junctional nevus immediately preceding the development of infiltrating melanocarcinoma”* shown in figure 9 in their 1953 article shows a melanoma in situ. From the current point of view, most of what has been published on the pathology of melanoma pre-1970s is only of historical interest. The articles, however, witness the different concepts of Ackerman, Allen, Spitz, and other pioneers of melanoma pathology, compared to our current view, particularly regarding melanoma in situ. What has been defined a precursor by Allen and Spitz, would be called a melanoma today.

In the early 1970s WH Clark and coworkers propagated a “*histogenetic*” classification of melanoma, which continues to be relevant until today [78,79]. In its original form the classification included 3 subtypes: nodular melanoma, superficial spreading melanoma, and lentigo maligna melanoma. Nodular melanoma was typified by pure vertical growth, while superficial spreading melanoma expands along the epidermis (radial or horizontal growth phase). The fourth subtype, acral lentiginous melanoma, was added later. Around the same time in the early 1970s, A Breslow introduced the invasion thickness as prognostic marker for primary skin melanoma [80,81], and in the mid-1970s, AB Ackerman and coworkers set forth histopathologic criteria for the diagnosis of melanoma that are still widely used by dermatopathologists (Table 1) [82]. Ackerman also popularized the concept of melanoma in situ, clinically and pathologically, and denied the concept of precursor lesions such as “Allen’s active junctional nevus”, “Hutchinson’s melanotic freckle”, “Kossard’s lentiginous dysplastic nevus of the elderly”, and “precancerous melanosis of Dubreuilh”. According to his opinion, these were evasions from the correct diagnosis of melanoma in situ.

In 1992 the National Institutes of Health (NIH) held a consensus conference to discuss the clinical and histological characteristics of early melanoma [83]. The panel of the consensus conference agreed that melanoma in situ is a distinct entity. With this official acceptance of “melanoma in situ” as authentic melanoma, the stage was finally set for early recognition to lift off. The increased public awareness, the availability of a new, accurate, and affordable in vivo examination technique, and the lower hesitancy of pathologists to diagnose melanoma in situ acted in accordance: The incidence for melanoma skyrocketed and increased more than for any other type of cancer.

Conventional pathology is still the gold standard for melanoma diagnosis but it is far from perfect. There is a large discrepancy of opinions and concepts among pathologists who tend to disagree on classification, terminology, the significance of subtypes, and on the model of tumor progression, but most importantly, they tend to disagree on the diagnosis [84–87]. For certain types of lesions there is large inter- and intra-observer variability among community-based pathologist whether a given lesion is benign or malignant. The most common issues of this sort concern the diagnosis of small or flat lesions and lesions with a spitzoid morphology. For these lesion categories, the community suggested terms with uncertain prognosis such as atypical Spitz tumor (AST) [88] and superficial atypical melanocytic proliferation of uncertain malignant significance (SAMPUS) [89]. There is certainly a need for such categories in practice but there are different views about the best way to express this ambiguity. One school of thought will blame the lesion (“the lesion does not know what it is”), the other the ignorance of the reporting pathologist (“the pathologist does not know what it is”).

In the early 2000s the molecular revolution in medicine gained momentum and new observations challenged our concepts of melanoma biology. The first turning point was the discovery of the significance of BRAF mutations in melanoma and in nevi [90]. This was soon followed by the detection of other tumorigenic mutations in other oncogenes [91] and climaxed in the description of the genomic landscape of melanoma [92]. While some of these discoveries translated into the identification of “druggable” biologic targets [93], the new insights into the genetic landscape of melanoma did not translate into reliable diagnostic methods for borderline lesions. Although molecular techniques such as fluorescence in situ hybridization (FISH) [94] or comparative genomic hybridization (CGH) [95] have been used to better classify borderline lesions such as Spitz tumors, they remain auxiliary techniques, requiring an integration with clinical and dermoscopic observations as well as with conventional pathology[96].

The recent hype associated with AI and deep learning in image based diagnostic medicine did not leave dermatopathology untouched [97]. Using random crops of digitized whole slide scans, Hekler et al showed that an algorithm trained by deep learning was capable off differentiating melanoma from nevi as accurate as pathologists [98]. It is, however, currently unknown how such algorithms will perform in the everyday practice.

### Summary and Interpretation

There can be no doubt that the clinical, dermatoscopic, and histopathologic criteria for the diagnosis of melanoma changed significantly over time. New inventions such as dermoscopy, TBP, and new developments in the field of AI and molecular medicine continuously modify the way we diagnose melanocytic proliferations. These developments in conjunction with increased public awareness shifted the landscape of melanoma diagnosis towards an increased detection of borderline lesions, especially with early melanomas. In a few decades we passed from an era of significant underdiagnosis to overdiagnosis. By overdiagnosis we refer to the inflation of the diagnosis of in situ or microinvasive melanomas with unknown prognostic significance. The undesired consequences of overdiagnosis should not be taken lightly. Apart from putting a significant financial burden on health care systems, overdiagnosis is associated with increased anxiety and morbidity of affected individuals. However, the recent suggestion of Welch and coworkers, that we should stop performing biopsies for lesions smaller than 6 mm, indicates lack of knowledge of current diagnostic techniques such as dermatoscopy. Some, albeit not all melanomas, can be diagnosed with confidence by dermatoscopy even when they are smaller than 6 mm (Figure 1). If early recognition of melanoma translates into improved survival is still a matter of debate. This question is not easy to answer. It would demand a randomized controlled trial with 2 arms. In 1 arm all lesions smaller than 6 mm that can be identified as melanomas by dermoscopy would be excised, in the other arm these lesions would be left alone until they reach the size of 6 mm. Since such a trial has not been performed and will not be completed in the near future, we have to rely on indirect evidence such as invasion thickness.

It is also true that early recognition has become a business. Feeding the business demands that the melanoma epidemic is constantly rising. However, to attribute the recent decline of melanoma mortality solely to the invention of new therapies is a slap in the face of all clinicians who dedicated their work to early recognition. Dermatologists or primary care clinicians, who work on the forefront of early diagnosis, are not greedy businessmen who stir up and exploit anxiety only for their own profit, in the same way as basic researchers and the pharmaceutical industry, who invent and develop new treatments against cancer, are not altruistic cure-alls.

Instead of turning back the wheel of time and ignoring the innovations of the last 30 years the inflated melanoma epidemic is best tackled otherwise. First, like any other diagnostic technique dermatoscopy needs training and expertise and it can have undesired side effects if used by inexperienced users. Better training will produce better dermatoscopists, who know the limitations of the technique and will make better decisions. If used appropriately by sufficiently trained and experienced clinicians, dermatoscopy will reduce and not inflate the number of excisions and biopsies. Second, pathologists who sign out melanocytic lesions need specific training in clinical dermatology. They need to be aware that borderline lesions are best diagnosed with an integrated approach taking into account clinical, dermatoscopic and, in some cases, also molecular findings. Third, clinicians and pathologist should not be paranoid of missing a melanoma. Overdiagnosis should be as undesirable as underdiagnosis. In some parts of the world vulnerability to malpractice lawsuits leads to over anxiousness, which leads to excessively low thresholds and overdiagnosis. Forth, we need a shift of policy with regards to incentives. Reimbursements for monitoring techniques such as TBP or digital dermatoscopy should be in the range of excisions. Reimbursement of clinicians, who need a disproportionally large number of biopsies to detect one melanoma, should be capped. Fifth, slides of pathology labs with a disproportionally large number of melanoma diagnoses should be reviewed by an expert panel. If the panel concludes that the threshold for melanoma diagnosis is below current standards, pathologists should be offered retraining.

I acknowledge that some of these suggestions will be unpopular among dermatologists and pathologists. There is, however, no other way to restore the trust in the accuracy of melanoma diagnosis. Without this trust, all efforts directed towards early recognition of melanoma will be in vain.

## Figures and Tables

**Table 1:** Significant histopathologic features of superficial spreading melanoma according to Price, Rywlin, and Ackermann 1976

Poor circumscription of the intraepidermal melanocytic component of the lesion with lateral extension of individual melanocytes
Increased number of melanocytes, solitary and in nests, within and above the epidermal basal-cell layer and within adnexal epithelium (pagetoid appearance)
Marked variation in size and shape of the melanocytic nests
Confluence of melanocytic nests rather than discrete nests.
Absence of maturation of melanocytes with descent into the dermis.
Melanocytes with nuclear atypia
Melanocytes in mitosis
Necrosis or degeneration of melanocytes
